# Early Return to Competition After vNOTES Ovarian Cystectomy in a Professional Athlete: A Case Report

**DOI:** 10.7759/cureus.100078

**Published:** 2025-12-25

**Authors:** Sotaro Hayashi, Satoshi Nishiyama, Eriko Iito, Tomohito Kobiyama, Naho Tokunaga, Koki Yagi, Mao Sekimata, Naoki Abe, Sachino Kira, Chiho Kadota, Masamitsu Kurakazu, Lifa Lee, Hiroshi Tsujioka

**Affiliations:** 1 Obstetrics and Gynecology, Iizuka Hospital, Iizuka, JPN

**Keywords:** early recovery after surgery, minimally invasive surgery, ovarian cystectomy, professional athlete, surgical case reports, vnotes

## Abstract

There are currently no studies that have evaluated return-to-sport or return-to-competition outcomes in professional athletes following gynecologic surgery. In addition, reports describing return to competition after vaginal natural orifice transluminal endoscopic surgery (vNOTES) in professional athletes are extremely limited. The present report focuses on early return to competition following vNOTES ovarian cystectomy in a professional athlete.

We report the case of a 20-year-old nulliparous professional bowler who underwent vNOTES ovarian cystectomy for a 9-cm left serous cystadenoma. The operation time was 66 minutes with minimal blood loss, and the patient was discharged on postoperative day three. She resumed training on postoperative day four, became asymptomatic by day 10, and returned to a national competition on postoperative day 23, finishing fourth.

This case demonstrates an exceptionally rapid postoperative recovery and represents one of the earliest documented returns to competition following gynecologic adnexal surgery, suggesting that vNOTES may offer distinct advantages for athletes who require preservation of abdominal wall integrity and rapid restoration of core function.

## Introduction

Minimally invasive surgery is the standard treatment for benign ovarian tumors. In conventional laparoscopy, transabdominal port placement is required, which can result in postoperative pain, muscle injury, and delayed recovery. For professional athletes, whose performance depends heavily on abdominal and core muscle function, abdominal wall trauma may significantly delay return to sport.

Vaginal natural orifice transluminal endoscopic surgery (vNOTES) is a minimally invasive surgical approach that enables access to the peritoneal cavity through the vagina, thereby avoiding abdominal wall incisions. This technique is increasingly recognized in gynecology for its potential to reduce postoperative pain and facilitate faster functional recovery. vNOTES has been reported in randomized controlled trials and systematic reviews to provide superior outcomes compared with laparoscopy, including reduced postoperative pain, shorter hospital stays, and faster return to daily activities [1-4].

However, how these advantages translate to athletic populations remains largely unexamined. In gynecologic surgery, no studies have evaluated return-to-sport or return-to-competition outcomes. Because vNOTES causes no abdominal wall trauma, it may offer greater benefits than laparoscopy for athletes, whose performance depends on core stability. Nevertheless, empirical data to support this concept are lacking.

Here, we present a case of a professional athlete who underwent vNOTES ovarian cystectomy and achieved an exceptionally rapid return to competition, providing the first sport-specific recovery insight in the context of gynecologic minimally invasive surgery.

## Case presentation

A 20-year-old nulliparous professional bowler presented with lower abdominal pain. She competed at the national level, and her sport required repetitive trunk rotation and core muscle engagement. She had no notable past medical, surgical, or family history. Physical examination revealed a soft, non-tender abdomen, and bimanual pelvic examination identified a mobile, enlarged mass in the lower abdomen, estimated to be around the size of a large hen’s egg.

**Figure 1:**
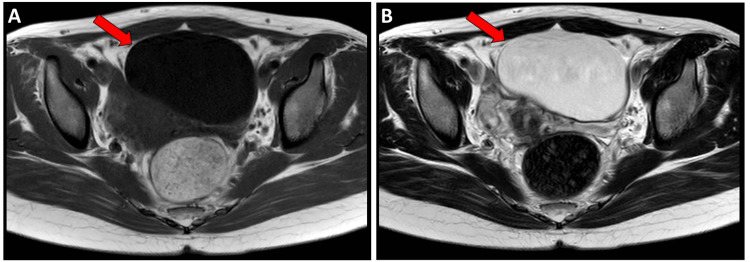
Pelvic magnetic resonance imaging findings. (A) T1-weighted image showing a unilocular cystic mass arising from the left ovary (arrow). (B) T2-weighted image demonstrating a high-intensity cystic lesion corresponding to the same ovarian mass (arrow).

Transvaginal ultrasonography revealed a unilocular cystic lesion in the left adnexal region measuring 72 × 58 mm, with benign features. Pelvic magnetic resonance imaging (MRI) confirmed a unilocular cyst arising from the left ovary measuring 92 × 72 × 60 mm, showing low signal intensity on T1-weighted MRI and high intensity on T2-weighted MRI (Figure 1).

Tumor markers were within normal ranges, except for mild elevation of carbohydrate antigen 19-9 (152.4 U/mL). Preoperative laboratory findings are summarized in Table 1.

**Table 1:** Preoperative laboratory findings

Parameter	Value	Reference range
White blood cell count (/µL)	4170	3300-8600
Hemoglobin (g/dL)	12.6	11.6–14.8
Platelet count (×10⁴/µL)	25.1	15.8–34.8
C-reactive protein (mg/dL)	0.02	<0.14
Aspartate aminotransferase (U/L)	18	13–30
Alanine aminotransferase (U/L)	14	7–23
Creatinine (mg/dL)	0.54	0.46–0.79
Carcinoembryonic antigen (ng/mL)	1.5	0-5
Carbohydrate antigen 19-9 (U/mL)	152.4	<37
Cancer antigen 125 (U/mL)	10.4	<35

**Figure 2:**
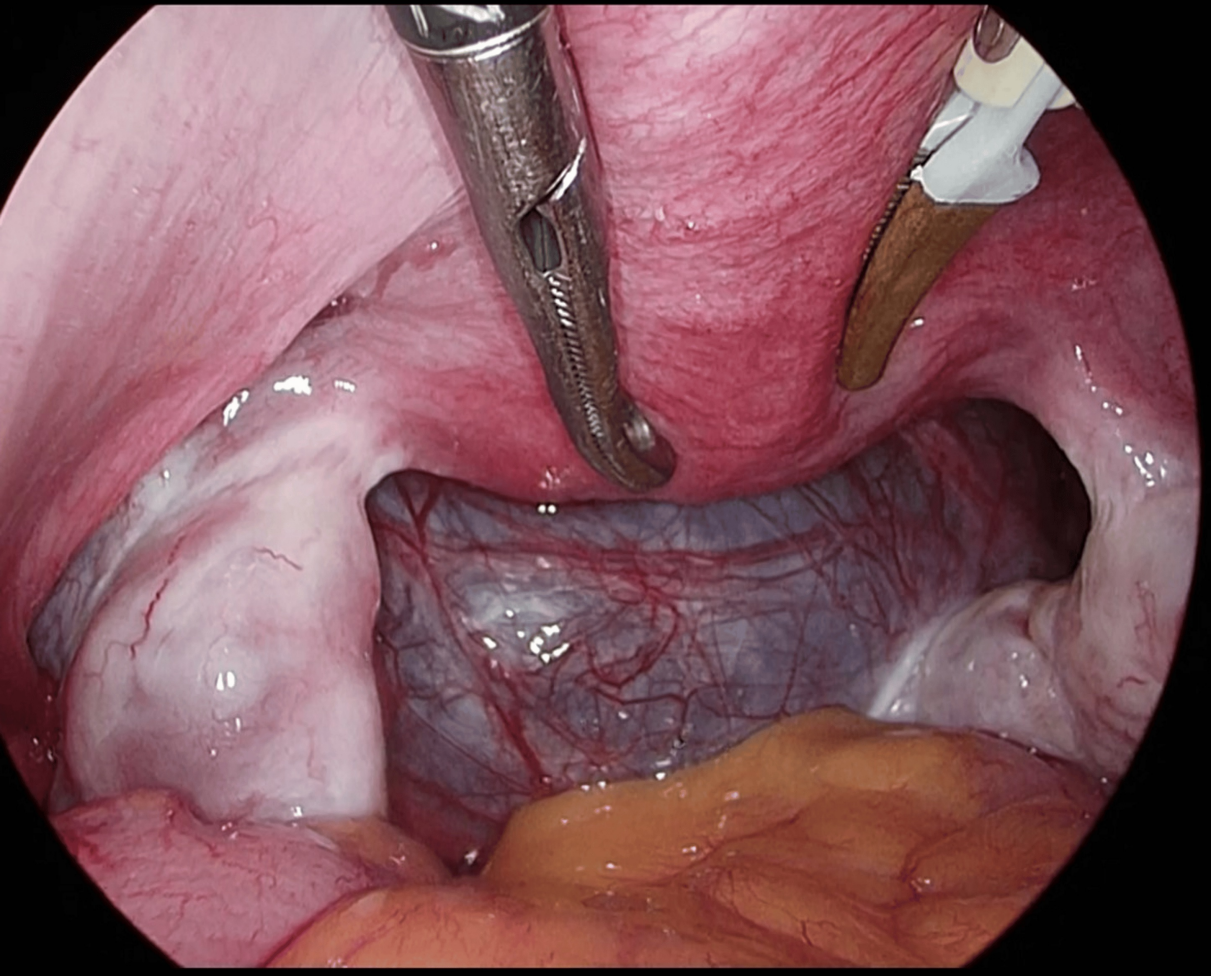
Intraoperative view of the pelvic cavity observed through the transvaginal laparoscopic approach during the vNOTES procedure. vNOTES, vaginal natural orifice transluminal endoscopic surgery. vNOTES: Vaginal natural orifice transluminal endoscopic surgery

The preoperative diagnosis was serous cystadenoma of the left ovary. Surgery was indicated based on the size of the cyst. Considering that the patient was scheduled to participate in a national bowling tournament within one month, vNOTES ovarian cystectomy was chosen to avoid abdominal incisions and preserve athletic performance. The procedure was performed under general anesthesia. The peritoneal cavity was accessed transvaginally (Figure 2).

**Figure 3:**
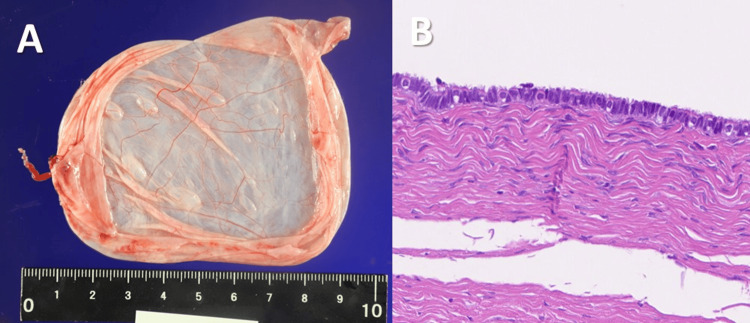
Gross specimen of the excised ovarian cyst (A). Histopathological examination revealed that the cystic wall was lined by a single layer of cuboidal epithelium, consistent with serous cystadenoma.

The left ovary was enlarged to the size of a large hen’s egg, and the cyst was carefully exteriorized through the vagina. The cyst wall was punctured extracorporeally, and its contents were aspirated; extracorporeal cystectomy with ovarian reconstruction was completed without complications. The operation time was 66 minutes with minimal blood loss. The specimen was a thin-walled cyst (Figure 3), and histopathology confirmed a benign serous cystadenoma.

Postoperatively, the patient reported minimal pain, was ambulating independently on postoperative day one, and was discharged on postoperative day three. She resumed training on postoperative day four with only mild discomfort, with no limitation in performance. By postoperative day 10, she was completely asymptomatic, and by day 23, she competed in a national tournament, placing 4th.

## Discussion

Multiple randomized controlled trials and systematic reviews have demonstrated that vNOTES offers several advantages over conventional laparoscopy, including reduced postoperative pain, shorter hospitalization, and decreased analgesic requirements [1-4]. A series of 1,000 cases also showed low complication rates and high reproducibility [5].

However, in the field of gynecology, no studies have evaluated postoperative return-to-sport (RTP) or return-to-competition outcomes. Existing literature addresses only return to daily activity, and no data describe the recovery of sport-specific function in athletes.

In contrast, the sports medicine literature includes data on postoperative return to competition after minimally invasive surgery. In a large series of athletes undergoing laparoscopic repair for inguinal disruption, Piozzi et al. reported that 94.4% of athletes returned to full sports activity within approximately four weeks [6]. Their findings indicate that preservation of the abdominal wall and minimizing postoperative pain are critical determinants of athletic recovery.

In the present case, the patient resumed training on postoperative day 4 and returned to competition on day 23. This recovery rate is comparable to, or even faster than, that reported in sports-related laparoscopic procedures. Because vNOTES completely avoids abdominal wall incisions, it may offer superior recovery potential for athletes whose performance relies on core muscle function.

Although this is a single case and its generalizability is limited, it provides the first academic insight into return-to-sport outcomes following gynecologic surgery and highlights an important but previously unexplored clinical dimension. Long-term follow-up data were not available in the present case. In addition, postoperative scarring or adhesion formation could not be assessed, as no second-look surgery or imaging specifically evaluating adhesions was performed.

## Conclusions

vNOTES ovarian cystectomy enabled extremely rapid functional recovery and early return to competition in a professional athlete. In this case, the patient resumed training on postoperative day 4 and returned to national-level competition on postoperative day 23, representing an exceptionally early recovery trajectory following gynecologic adnexal surgery.

In the absence of evidence evaluating return-to-sport or return-to-competition outcomes after gynecologic surgery, this case highlights the potential clinical value of vNOTES for athletes who require preservation of abdominal wall integrity and rapid restoration of core function. Further studies with larger cohorts are warranted to systematically assess sport-specific recovery outcomes following gynecologic surgery, as the present findings are based on a single case.

## References

[1] Baekelandt JF, De Mulder PA, Le Roy I (2019). Hysterectomy by transvaginal natural orifice transluminal endoscopic surgery versus laparoscopy as a day-care procedure: A randomised controlled trial. BJOG.

[2] Baekelandt J, De Mulder PA, Le Roy I (2021). Adnexectomy by vaginal natural orifice transluminal endoscopic surgery versus laparoscopy: Results of a first randomized controlled trial (NOTABLE trial). BJOG.

[3] Housmans S, Noori N, Kapurubandara S (2020). Systematic review and meta-analysis on hysterectomy by vaginal natural orifice transluminal endoscopic surgery (vNOTES) compared to laparoscopic hysterectomy for benign indications. J Clin Med.

[4] Marchand GJ, Masoud AT, Ulibarri H (2024). Systematic review and meta-analysis of vaginal natural orifice transluminal endoscopic surgery versus laparoscopic hysterectomy. AJOG Glob Rep.

[5] Mone F, Mulcahy C, Navaratnam K, Phelan MM, Breathnach F, Alfirevic A, McAuliffe Frcog FM (2021). Assessing measures of aspirin adherence in pregnancy: Secondary analysis of the TEST randomized controlled trial. Eur J Obstet Gynecol Reprod Biol.

[6] Piozzi GN, Cirelli R, Salati I (2019). Laparoscopic approach to inguinal disruption in athletes: a retrospective 13-year analysis of 198 patients in a single-surgeon setting. Sports Med Open.

